# Enhanced Coagulation-Flocculation Performance of Iron-Based Coagulants: Effects of PO_4_^3-^ and SiO_3_^2-^ Modifiers

**DOI:** 10.1371/journal.pone.0137116

**Published:** 2015-09-04

**Authors:** Wei Chen, Huaili Zheng, Houkai Teng, Yili Wang, Yuxin Zhang, Chuanliang Zhao, Yong Liao

**Affiliations:** 1 Key laboratory of the Three Gorges Reservoir Region’s Eco-Environment, State Ministry of Education, Chongqing University, Chongqing, China; 2 National Centre for International Research of Low-carbon and Green Buildings, Chongqing University, Chongqing, China; 3 National Research Center of Industrial Water Treatment Engineering and Technology, Tianjin Chemical Research and Design Institute, Tianjin, China; 4 Research Center for Water Pollution Source Control and Eco-remediation, Beijing Forestry University, Beijing, China; 5 State Key Laboratory of Urban and Regional Ecology, Research Center for Eco-Environmental Sciences, Chinese Academy of Sciences, Beijing, China; Monash University, AUSTRALIA

## Abstract

PO_4_^3-^ and SiO_3_^2-^ are often used as modifier to improve stability and aggregating ability of the iron-base coagulants, however, there are few reports about their detailed comparison between the coagulation performance and mechanisms. In this study, three coagulants—polyferric phosphoric sulfate (PFPS), polysilicon ferric sulfate (PFSS), and polyferric sulfate (PFS) were synthesized; their structure and morphology were characterized by Fourier transformed infrared (FT-IR) spectroscopy, X-ray diffraction (XRD) and Scanning electron microscope (SEM). Alkali titration and Ferron species analysis were employed to investigate the hydrolysis performance and species distribution. Jar test was conducted to measure their coagulation behaviors at different dosage, pH, and temperatures in which the flocs properties were measured. The results showed that a number of new compounds were formed due to the presence of PO_4_^3-^ and SiO_3_^2-^. Moreover, PFPS and PFSS had similar level in Fe_a_ as well as Fe_b_. Among them, PFPS produced more multi-core iron atoms polymer and content of Fe_b_, and the formed flocs were larger and denser. It exhibited superior coagulation performance in terms of turbidity reduction, UV_254_ removal and residual ferric concentration. Jar test and floc breakage/regrowth experiments indicated other than charge neutrality, the dominated mechanism involved in PFSS was the adsorption between polysilicic acid and solution particle, while PFPS was sweeping, entrapment/adsorption resulting from larger polymer colloid of Fe-P chemistry bond.

## Introduction

Coagulation-flocculation is a widely used technology which separates impurities from polluted water bodies. Coagulants typically adopted for water treatment are inorganic iron and aluminum salts [1–3]. In addition to Al-base coagulants, Fe-based coagulants such as Fe_2_(SO_4_)_3_ and FeCl_3_ have been increasingly favored due to their non-toxicity, larger flocs size, superior turbidity reduction, and DOC removal efficiency [4–6]. However, inherent, strong and uncontrolled hydrolysis properties of iron (III) ion results in lower polymerization of Fe-based coagulants and inferior coagulation performance. Recently, researchers have begun to focus more on high molecular pre-polymerized Fe-based coagulants, which benefit enhanced charge neutralization ability and molecular size (weight), as well as aggregation power [7–9].

Compared to conventional Fe-based coagulants, the introduction of organic or inorganic modifiers improves the performance of coagulants significantly. Various types of additives have been proposed, in which polysilicic acid and PO_4_^3-^ have received the most attention [10–12]. Results of previous research indicate that polysilicic acid linked via silicon–oxygen–silicon bonds, plus various acidified polysilicate species such as Si_2_O_3_(OH)_4_^2−^ and Si_3_O_5_(OH)_5_^3−^, added to the structure of Fe-based coagulants can prevent iron species from precipitating and successfully increase molecular weight and size [2, 13]. Zouboulis et al. prepared a pre-polymerized inorganic coagulant, such as polyferric silicate sulphate (PFSiS) under different Fe/Si and OH/Fe molar ratios. The result showed that the introduction of polysilicic acid into the structure of the coagulants increased the molecular size and enhanced the aggregating power of the coagulants and further improved the water treatment efficiency [13]. Wang et al. studied a series of tailor-made polyferric silicates (PFSiS), which was prepared by adding various polysilicic acid species as modifiers. The experiments confirmed that polysilicic acid addition increased bridge effect and slowed the formation of Fe(OH)_3_(s), resulting in enhanced the coagulation performance, as well [2].

In addition to polysilicic acid, PO_4_^3-^ could react with Fe^3+^ to synthetize chemical Fe-P bonds. The complexation of Fe-P bonds facilitates the formation of high polymer with improved stability and aggregating efficiency [14, 15]. Tang et al. prepared PPFC using phosphate as a stabilizer, and proved that introduction of PO_4_^3-^ improves the stability of Fe-based coagulants and markedly improve coagulation performance [16]. Zhu et al. synthesized a composite coagulant, Polymeric aluminum ferric sulfate (PAFS), with FeSO_4_·7H_2_O and H_3_PO_4_. PAFS showed better coagulating effects in terms of charge neutralization, turbidity, and COD reduction [17, 18]. Though much attention had been given to polyferric sulfate (PFS) combinations containing polysilicic acid and PO_4_^3-^, research which compares the two modified coagulants in terms of characterization, coagulation performance, floc properties, and coagulation mechanism is yet lacking. In effort to remedy the shortage, this study conducted a detailed investigation into the distinct mechanisms, properties, and water treatment efficiency of two modified coagulants. (PFS without additives was used as a comparative reference.)

In this study, three coagulants were synthesized using FeSO_4_·7H_2_O and NaClO_3_ as raw materials. Two additives, PO_4_^3-^ and polysilicic acid, were introduced as modifiers to prepare composite coagulants polyferric phosphoric sulfate (PFPS) and polysilicon ferric sulfate (PFSS), respectively. The structure and morphology of three coagulants were investigated by Fourier transformed infrared (FT-IR) spectroscopy, X-ray diffraction (XRD), and scanning electron microscopy (SEM). Fe species distribution of various products was also studied by ferron and alkali titration measurements. A series of jar test experiments were conducted to form a detailed comparison of the three coagulants as far as coagulation–flocculation behavior according to variations in dosage, initial water pH, and temperature. The flocs properties of various coagulants were also comparatively researched in terms of growth, strength, and regrowth in the coagulation of synthetic kaolin-humic acid water samples. Besides, the primary coagulation mechanism of each coagulant is discussed in detail, in conjunction with its coagulation performance and floc properties. After which, a series of comparative experiments with commercial coagulants were conducted.

## Materials and Methods

### Preparation of coagulants

In this study, three Fe-based coagulants were prepared and comparatively investigated in the laboratory. 55.6g of FeSO_4_•7H_2_O (A.R.) was dissolved in 45ml 20% H_2_SO_4_ solution under slow stirring in a beaker to obtain a homogeneous ferrous sulfate liquid mixture, the temperature of which was set to 60°C by a thermostatic water bath. 3.55g NaClO_3_ (A.R.) was added into the reaction vessel to oxidize the Fe(II) in pickle liquor into Fe(III). After preparing the Fe(III) solution, the three coagulants were obtained according to different methods listed as follows.

#### a. PFS

In this study, NaHCO_3_ was used to adjust the basicity and further promote the polymerization of the coagulant. When the NaHCO_3_ was added into acid solution, the balance of H^+^ and OH^-^ ions would be disturbed, and the concentration of OH^-^ ions increased. The hydroxide ion will replace the sulphate ion in the hydrolysis stage and therefore, the polymerisation will occur as follows:

$${\text{Fe}}_{2}{\left({\text{SO}}_{4}\right)}_{3}+{\text{nOH}}^{-}\to {\text{Fe}}_{2}{\left(\text{OH}\right)}_{\text{n}}{\left({\text{SO}}_{4}\right)}_{3-\text{n}/2}+\text{n}/2{\text{SO}}_{4}{}^{2-}$$ (Step 1. Hydrolysis)

$${\text{mFe}}_{2}{\left(\text{OH}\right)}_{\text{n}}{\left(\text{SO}4\right)}_{3-\text{n}/2}\to {\left[{\text{Fe}}_{2}{\left(\text{OH}\right)}_{\text{n}}{\left({\text{SO}}_{4}\right)}_{3-\text{n}/2}\right]}_{\text{m}}$$ (Step 2. Polymerisation)

Step 1 and Step 2 is the simplified scheme of hydrolysis-polymerisation process, proposed by Dousma and Bruyn (1978) [5, 19]. In the experiment, 5.04g NaHCO_3_ (A.R.) powder was added into the Fe(III) solution under the slow stirring at the rate of 95–100 rpm in 60°C thermostatic water bath. After stirring for 60 min, the reaction mixture was stored at room temperature for further polymerization and aging.

#### b. PFSS

Water glass (modulus: 2.33 C.R.) was diluted to 3.0% (m/m) with de-ionized water, then introduced into sulfuric acid solution (20.0% m/m) slowly with a magnetic stirring apparatus at room temperature (25°C). The pH was regulated to 3.0±0.2 by 0.5M H_2_SO_4_ and 1M NaOH. After that, silicic acid solution of around 2.5% was kept steady and aged for about 1h for effective polymerization. A measured amount of polysilicic acid (Si/Fe = 0.2) was added to the metal salt solution at slow speed and stirred. After the addition of silicic acid for 10 min, the remaining preparation steps of adding NaHCO_3_ were the same as for PFS.

#### c. PFPS

In order to obtain the coagulants properties and coagulation efficiency of PFS, PFSS and PFPS at the same conditions as accurately as possible, it is necessary to keep the basicity of three coagulants consistent in the preparation process. Moreover, the logarithm value of first dissociation constant (pK_1_) of H_3_PO_4_ is 2.12, therefore the pH could remain unchanged with the addition of NaH_2_PO_4_ during the preparation of PFPS [20, 21]. Thus the NaH_2_PO_4_ was selected in this research. In the experiment, 6.24g NaH_2_PO_4_ was dissolved in 20ml of de-ionized water, after which the solution was added to the reaction vessel through a peristaltic pump. After the NaH_2_PO_4_ solution was completely added, the remaining preparation steps of adding NaHCO_3_ were the same as for PFS.

The main physic-chemical properties of three prepared coagulants samples are shown in Table 1.

**Table 1:** Main physic-chemical properties of the prepared coagulant samples. pH_1_, pH_15_ and pH_30_ represent the pH value at aging time of 1, 15 and 30 days, respectively.

Coagulant	Concentration of total iron (%)	Density (g/cm^3^)	Zeta (1% water solution)	pH_1_ (1% water solution)	pH_15_ (1% water solution)	pH_30_ (1% water solution)	Basicity	Stability
PFS	11.37	1.45	2.99	2.26	2.21	2.11	9.12	17^a^
PFSS	8.93	1.09	1.79	2.53	2.49	2.46	10.29	44^b^
PFPS	11.55	1.41	2.67	2.25	2.22	2.23	10.34	—

^a^ Destabilized time (day).

^b^ Gel effect time (day).

—No yellow precipitate or gel-like substance appeared within two months.

### Structure and morphology

The liquid coagulant samples were dried in a vacuum oven at 70 C for several days then the dried solids were ground into powder by mortar and pestle. The prepared samples were measured by D/Max-3C X-ray diffractometer with Cu K radiation in the range of 5°—65°(2θ) at a scan rate of 4°/min to obtain the X-ray diffraction (XRD) of each product. The FT-IR characteristic peaks were obtained by measuring the samples with FT-IR spectrophotometer (IR Prestiger-21, Japan) in the range of 4000–400 cm^−1^. The morphologies of the coagulants were also observed by scanning electron microscope (SEM).

### Alkali titration analysis

In order to indirectly measure the hydrolytic polymerization capability and amount of polymer species of coagulants throughout the coagulation-flocculation process, a series of experiments were performed to test the effect of alkali content on the dynamic trends of the pH value of the coagulant solution [18]. First, 2ml 2.8mol/l Fe-based liquid coagulant was diluted to 40ml with 20°C double-distilled water in 100ml glassware. The 1mol/l NaOH was titrated into the solution at a titration rate of 1ml/min while stirred magnetically. For each titration, the pH of the solution was recorded every 0.1ml NaOH.

### Ferron and turbidity reduction analysis

Fe species can be artificially divided into three distinct types, Fe_a_, Fe_b_, and Fe_c_, based on the extent of hydrolysis and polymerization taking place in the mixed solution. Fe_a_ represents the free ion and mononuclear hydroxyl complex, while Fe_b_ is defined by the formation of many poly-nuclear complexes. Fe_c_ comprises of complexes of high molecular polymer and Fe(OH)_3_ [22, 23]. For the purpose of classifying the Fe species suspensions appropriately, the ferron-complexation timed spectrophotometric method was adopted, based on the standard adsorption curves of the reaction between Fe-based coagulants and ferron reagents (8-hydroxy-7-iodoquinoline-5-sulphonic acid). Visible light absorbance was measured as a function of time at a wavelength of 597 nm to quantify the amount of Fe complexes. Absorbance observed within the first minute of the liquor was ascribed to Fe_a_, and to the sum of Fe_a_ and Fe_b_ four hours later when the absorbance ended in a plateau. After boiling for 30 min, the absorbance was marked as unread Fe_-_, and Fe_c_ value was calculated by subtracting Fe_a_ and Fe_b_ from the Fe_-_. (This experimental method was based on a previous paper by Dong [24, 25].)

### Coagulation experiments

#### Synthetic test water

Standard stock solution of 1g/l humic acid was prepared by dissolving 1g humic acid and 0.04g NaOH into 1 l de-ionized water under magnetic stirring for 1 h. 2g kaolin was added to 1 l tap water and magnetically stirred for 30 min. After setting for 1h, the upper suspension was collected to regulate the turbidity of the synthetic test water sample. Synthetic humic acid–kaolin simulation water was prepared by diluting the HA stock solution with proportionally dechlorinated tap water and upper kaolin suspension to make 10 mg/l HA with turbidity of 15.0 ± 0.5 NTU. The initial desired pH value was adjusted using 0.2 M NaOH and 0.1 M H_2_SO_4_. Characteristics of synthetic water were presented in Table 2.

**Table 2:** Characteristics of synthetic water.

Turbidity (NTU)	UV_254_ (cm^−1^)	Zeta potential (mv)	pH
15±0.5	0.390 ± 0.015	−21.1 ± 0.5 Mv	8.05 ± 0.05

#### Coagulation test

Standard coagulation tests were conducted on a program-controlled jar test apparatus (ZR4–6, Zhongrun Water Industry Technology Development Co. Ltd., China) in an air-conditioned room. Each beaker contained 500 ml synthetic simulation water plus a predetermined coagulant dose, added by rapid stirring. For purpose of exploring the effect of PO_4_^3-^ and SiO_3_^2-^ modifiers on the coagulation-flocculation behaviors of Fe-based coagulants as accurately as possible, no any polyelectolyte was introduced. During each experiment, the coagulation process composed of a rapid agitation stage, at 250 rpm for 1 min, to obtain homogeneous dispersion, followed by a slow agitation stage, 40 rpm for 10 min, to aggregate the colloidal particles. Sedimentation was then encouraged for 30 mins [18, 26]. The zeta potential of combined particles was directly measured using a Zetasizer 3000Hsa, collected in water samples as soon as the rapid agitation stage finished. 150 ml supernatant sample was extracted from the beakers by a syringe 1 cm below the water surface, then were prefiltered using 0.45 μm cellulose acetate membrane before testing their UV_254_ (UV/VIS spectrophotometer) and residual iron concentration (atomic absorption spectrophotometer, Perkin Elmer, model 3110). The experiment was performed in triplicate, and the resultant value corresponded to the means of the two closest results.

### Floc size measurement, breakage and regrowth

The evolution of floc size throughout the mixing and coagulation periods described above was monitored by laser diffraction instrument (Mastersizer 2000, Malvern, UK) at a room temperature of 20±1°C. The suspension water was drawn through the optical unit of the Mastersizer and placed back in the jar by a peristaltic pump at a flow rate of 1.5 l/h. Inflow and outflow tubes 5 mm in inner diameter were symmetrically positioned on the opposite side of the beaker at a depth just above the paddle. Size measurements were taken every 30 sec. The data was logged onto a PC automatically throughout the duration of the jar test. In order to study the effect of hydraulic conditions on the evolution of flocs, increasing shear force was introduced to break flocs after the slow stir was finished. 5 min of floc breakage was performed at stir speed of 200 rpm. After the breakage stage, slow stirring was reintroduced (40 rpm) to allow flocs reaggregation. (A similar monitoring technique was used in a previous study, as well [27–30].)

The floc growth rate was calculated according to the slope of the rapid growth region:

$$\text{Growth rate}=\frac{\Delta \text{size}}{\Delta \text{time}}$$ (1)

After the flocs breakage and reaggregation stages, the floc strength factor and recovery factor were used to compare the floc strength and recoverability:

$$\text{Strength factor}\;\left(S_{f}\right)=\frac{d_{1}}{d_{2}}\times 100\%$$ (2)

$${\text{Recovery factor}(\text{R}}_{\text{f}})=\frac{d_{3}-d_{2}}{d_{1}-d_{2}}\times 100\%$$ (3)

where d_1_ represents the floc size in the steady stage (before breakage), d_2_ is the floc size after breakage, d_3_ is the floc size after re-growth before the second steady phase. Strength factor (S_f_) is the ratio of floc size after and before breakage at a higher shear rate, which indicates resistance of the formed flocs to shear force. Recovery factor (R_f_) refers to the re-aggregation of flocs to form larger flocs again after being subjected to shear breakage.

## Results and Discussion

### Modifier vs. X-ray diffraction

**Fig 1:**
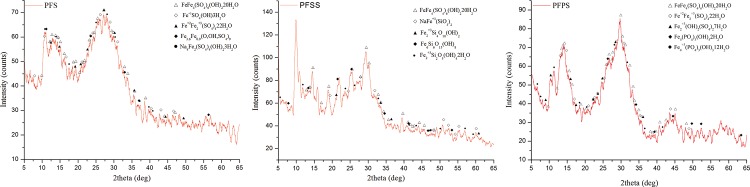
XRD pattern of PFS, PFSS and PFPS.

In order to identify the compounds/phases of products, the three coagulants were observed using X-ray powder diffraction. Results are shown in Fig 1.

The X-ray diffraction spectra of the coagulants were analyzed by Jade 6.0 software. Results indicated similar major FeFe_4_(SO_4_)_6_(OH)_2_20H_2_O phases in all three products at 2θ, of 4.799°, 9.574°, 12.932°, 16.650°, 25.502°, and 43.037°. Similar Fe^+2^Fe_2_^+3^(SO_4_)_4_22H_2_O and Fe^+3^(SO_4_)(OH) 5H_2_O crystals were also observed in each sample. Introduction of PO_4_^3-^ synthetized the crystal structure as Fe^+2^Fe_5_^+3^(PO_4_)_4_(OH)_5_4H_2_O and Fe^+3^Fe2^+3^(PO_4_)_2_(OH)_3_5H_2_O in the crystalline phases of PFPS and PFSS, and SiO_3_^2-^ synthetized a hexagonal crystal structure as NaFe^+3^(SiO_3_)_2_, (SiO_2_)_x_, and NaFe^+3^(SiO_3_)_2_. Iron sulfate salts were nonstoichiometric compounds, with basic structure that did not easily change as overall composition changed; whereas the presence of new matter without standard molecular formula, plus weak Fe_2_(SO_4_)_3_, Fe_2_O_3_, Fe(OH)_3_, and Fe_3_O_4_ peaks, indicated that such materials as Fe^3+^, SO_4_^2−^, H_2_O, SiO_3_^2-^, and PO_4_^3-^ were combined by polymerization to form new compounds which may not have been included in the gallery. All three coagulant samples showed very different crystal structures. PO_4_^3—^modified coagulants produced larger, multi-core iron atom polymer, which contributed stronger bridging, sweep entrapment ability among the flocs—the primary mechanisms at work during the coagulation-flocculation process [26, 31].

### Modifier vs. FT-IR spectroscopy

**Fig 2:**
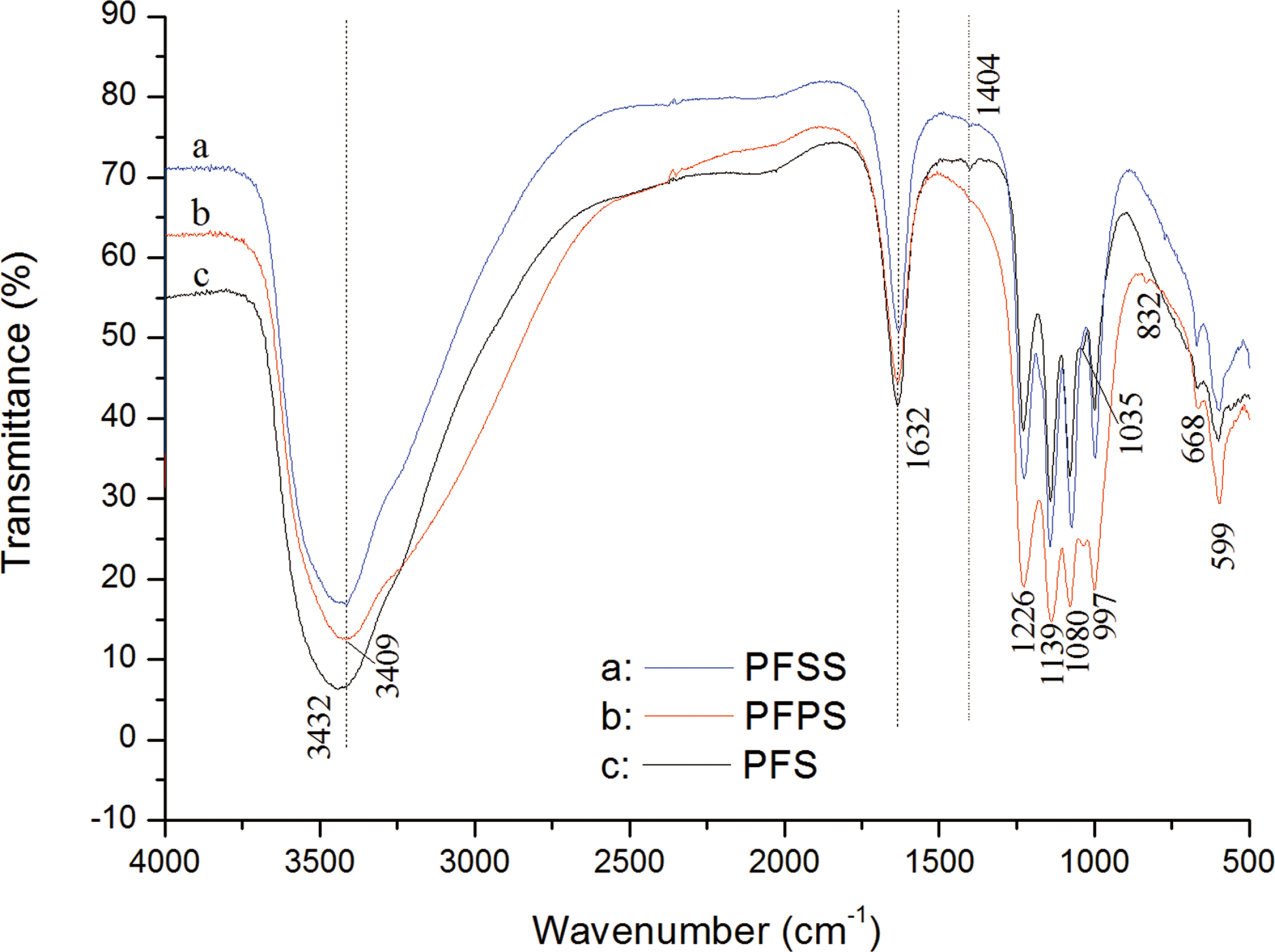
FT-IR spectroscopy of PFS, PFSS and PFPS.

The possible chemical bonds of three Fe-based coagulants were explored by FT-IR characteristic peaks in the range of 4000–400 cm−1. Fig 2 shows the FT-IR spectroscopy for PFS, PFSS and PFPS.

As shown in figure, strong absorption peaks were observed at around 3409 and 1632 cm^−1^, which were related to the stretching vibration of −OH groups and the H-O-H absorbed and crystallized in the coagulants [17]. It is worth noting that the wave number of the vibration of H-O-H kept consistent, while the wave number of -OH in PFSS and PFPS is slightly larger than that in PFS. The position shifts in the IR spectrum may be attributed to the spatial resistance of the nearby functional group resulted by the introduction of SiO_3_^2-^ and PO_4_^3-^. In addition, the characteristic absorption peak at 1035 cm^−1^ of PFS is assigned to the bending vibrations of Fe-OH-Fe. The absorption peak at 1035 cm^−1^ in FFPS was owing to the superposition of symmetrical and anti-symmetrical stretching vibration of -P = O groups [21]. The absorption peak is hardly observable in PFSS, the reason was attributed to that with the addition of SiO_3_^2-^, the Fe-OH-Fe bonds transformed to Si–O–Fe bond [32]. Moreover, the peak at 832 cm^−1^ corresponds to the symmetrical stretching vibrations of Fe–O–Fe group, which was weak or disappear in PFS and PFSS. The result suggests that the introduction of PO_4_^3-^ enhanced the stability of Fe-based coagulants. As a result, it is evident that the addition of SiO_3_^2-^ and PO_4_^3-^ has a significant role on the structure of Fe-based coagulants.

### Modifier vs. SEM morphology

**Fig 3:**
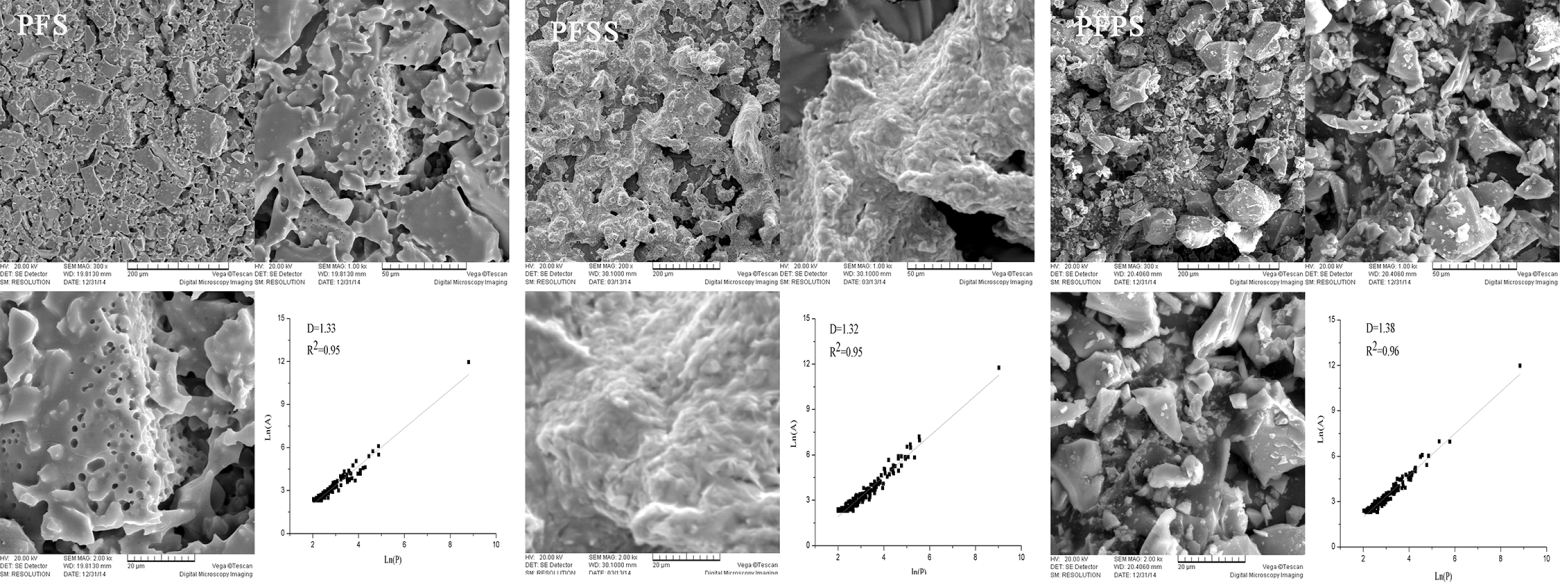
SEM photographs of PFS, PFSS and PFPS.

The morphologies of Fe-based coagulant samples prepared by introducing different anion modifiers were observed by SEM; results are presented in Fig 3.

As is clear in the images below of the coagulants’ respective microscopic structure (magnified 300, 1000 and 2000), the difference between the coagulant surface morphologies was significant. PFPS presented compact, irregular granule units, while the surface morphology of PFSS was wrinkled with three-dimensional, porous granules, and PFS exhibited porous, lamellar structures. Previous studies have shown that compact, irregular granule structure is more favorable as far as adsorbing and connecting colloidal particles and further enhancing the sedimentation of flocs [11, 18, 32].

The main reason for morphology differences among the three coagulants was the addition of various modifiers. The porous morphology of Fe-based coagulants was assigned to the hydrolysis of Fe ion. Compared to PO_4_^3-^, the effects of SiO_3_^2-^ in preventing iron ions from further hydrolysis was limited; but gel-like polysilicic acid was conducive to flocculating colloidal particles and forming larger flocs during the coagulation process [33, 34]. Additionally, the irregularity of solid products can be attributed to fractal dimension, which is closely related to solubility and coagulation efficiency [9, 18]. Some literatures pointed out that the coagulants with higher fractal dimensions exert positive effects on flocculation of colloidal particles and formation of bridge-aggregation among flocs [18, 35]. In this paper, fractal dimension is calculated using the linear correlation between the logarithms of perimeter (L) and area (A). Assisted by Image-Pro Plus 6.0 software, the average fractal dimensions of PFPS, PFSS, and PFS were determined to be 1.38, 1.32, and 1.33, respectively.

### Modifier vs. alkali titration curve

**Fig 4:**
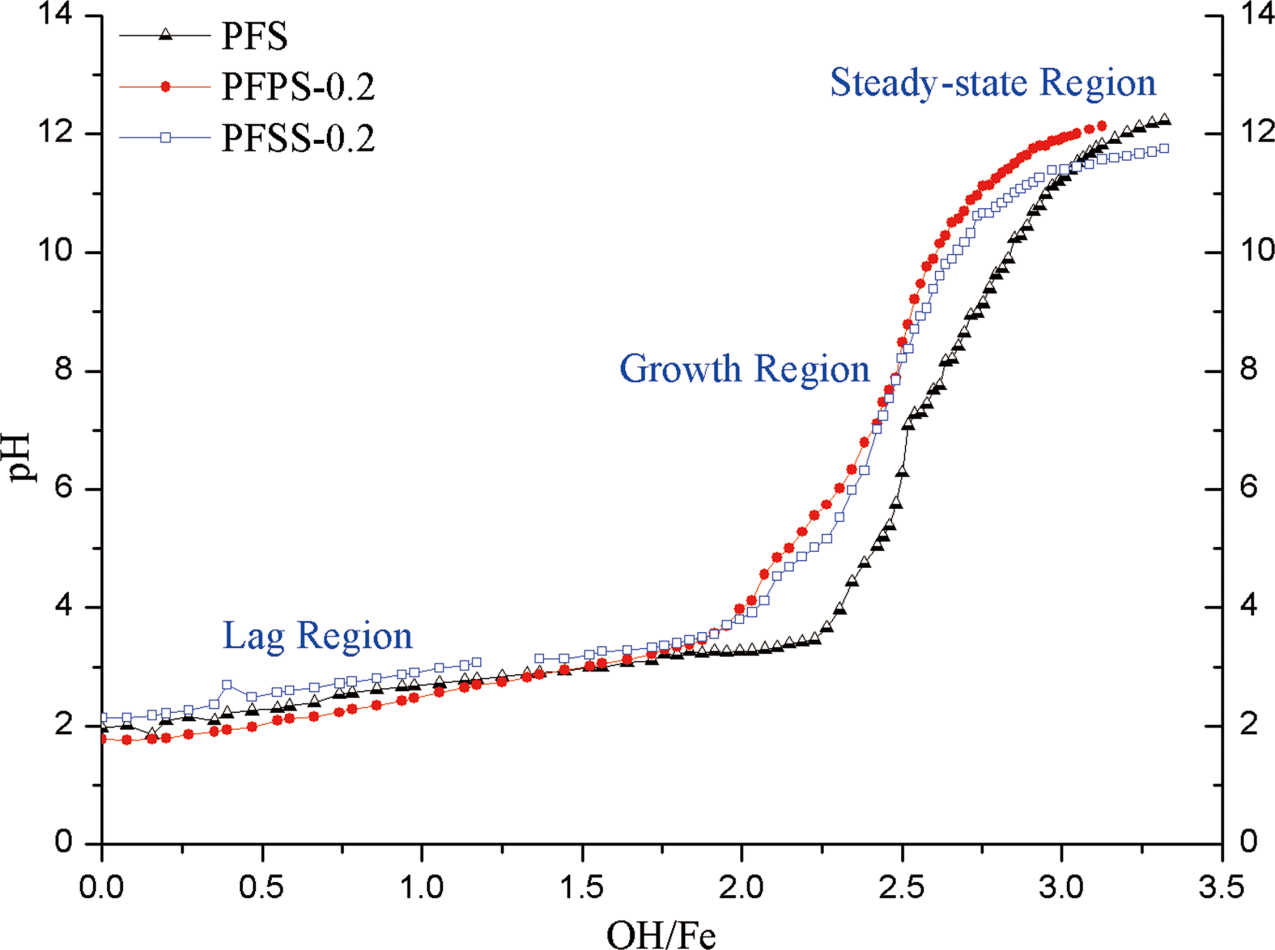
Alkali titration curve of three different coagulant.

In this paper, the hydrolytic polymerization performance of each coagulant was interpreted according to changes in pH value under alkali titration. Results are illustrated in Fig 4.

According to previous research, alkali titration includes three regions: lag, growth, and steady. In the lag region, alkali react with oligomer as Fe(OH)^2+^ and Fe_2_(OH)_2_^4-^. Reaction with the polymer and sedimentation correspond to the growth region and steady region, respectively [18, 36]. In the lag region, the order of pH value in this experiment was PFSS-0.2>PFS>PFPS-0.2, which can be ascribed to low ionization of polysilicic acid releasing fewer H^+^, while the complex of Fe^3+^ and PO_4_^3-^ resulted in lower pH value. The NaOH quantities titrated in the lag regions of PFSS and PFPS were less than that of PFS, because the addition of SiO_3_^2-^ and PO_4_^3-^ was conducive to the synthesis of more polymer species as illustrated in Fig 4 [5, 13, 37]. In the growth region, the curve rise rate of PFSS and PFPS was slower than PFS. This was caused by the alkali first reacting with the low polymer, then the high polymer, before sedimentation. In contrast, the quick reaction between NaOH and oligomer generated sedimentation immediately in PFS, producing the steep curve in the growth region. Basically, more polymer species, which favor better adsorption, bridging, and charge neutrality in the coagulation-flocculation process, were generated after introduction of PO_4_^3-^ and SiO_3_^2-^ modifiers [1, 23].

### Modifier vs. Fe species and turbidity reduction

**Fig 5:**
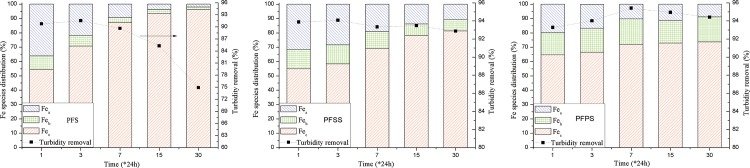
Fe species distribution and Turbidity reduction of three coagulants under various aging time.

The coagulation performance of Fe-based coagulants is closely related to the distribution of Fe species [25]. The Fe species distribution and turbidity reduction of PFS, PFSS, and PFPS under aging times of 1, 3, 7, 15, and 30 days were tested as illustrated in Fig 5. The content of Fe_c_ in PFS increased, while the Fe_a_ and Fe_b_ contents decreased between 1 to 30 days. At aging time over two weeks, a piece of faint yellow precipitate appeared in the bottom of the container, indicating a small amount of active ingredients in the solution. The turbidity reduction efficiency deteriorated with aging time, consistent with the species distribution inference. Compared to PFS, PFPS and PFSS exhibited higher Fe_a_ and Fe_b_ contents as aging time progressed, in relatively stable distribution. These results suggest that the introduction of PO_4_^3-^ and SiO_3_^2-^ modifiers enhanced the stability of the coagulants as well as their turbidity reduction. After PO_4_^3-^ and SiO_3_^2-^ were introduced into the Fe_2_(SO_4_)_3_ solution, Fe^3+^ reacted with PO_4_^3-^ and SiO_3_^2-^ to produce relatively stable Fe-P and Fe-Si bonds, resulting in more medium-weight polymers, which benefit stability and coagulation performance [17, 38]. After lengthier aging time, the Fe_b_ content in PFPS was higher than PFSS, and the turbidity reduction of PFPS was somewhat higher than PFSS. The small gap between PFSS and PFPS can be explained by the complexation of Fe^3+^ and PO_4_^3-^ favors good stability. In PFSS, the addition of polysilicic acid result in gel effect of coagulant after around 6 weeks aging, which further leading to the inconveniently application and deterioration of coagulation efficiency [2].

### Modifier and dosage vs. coagulation-flocculation performance

The structure, morphology, and species distribution of the samples are discussed above. A series of beaker experiments were also employed in order to test the water treatment performance of the coagulants.

**Fig 6:**
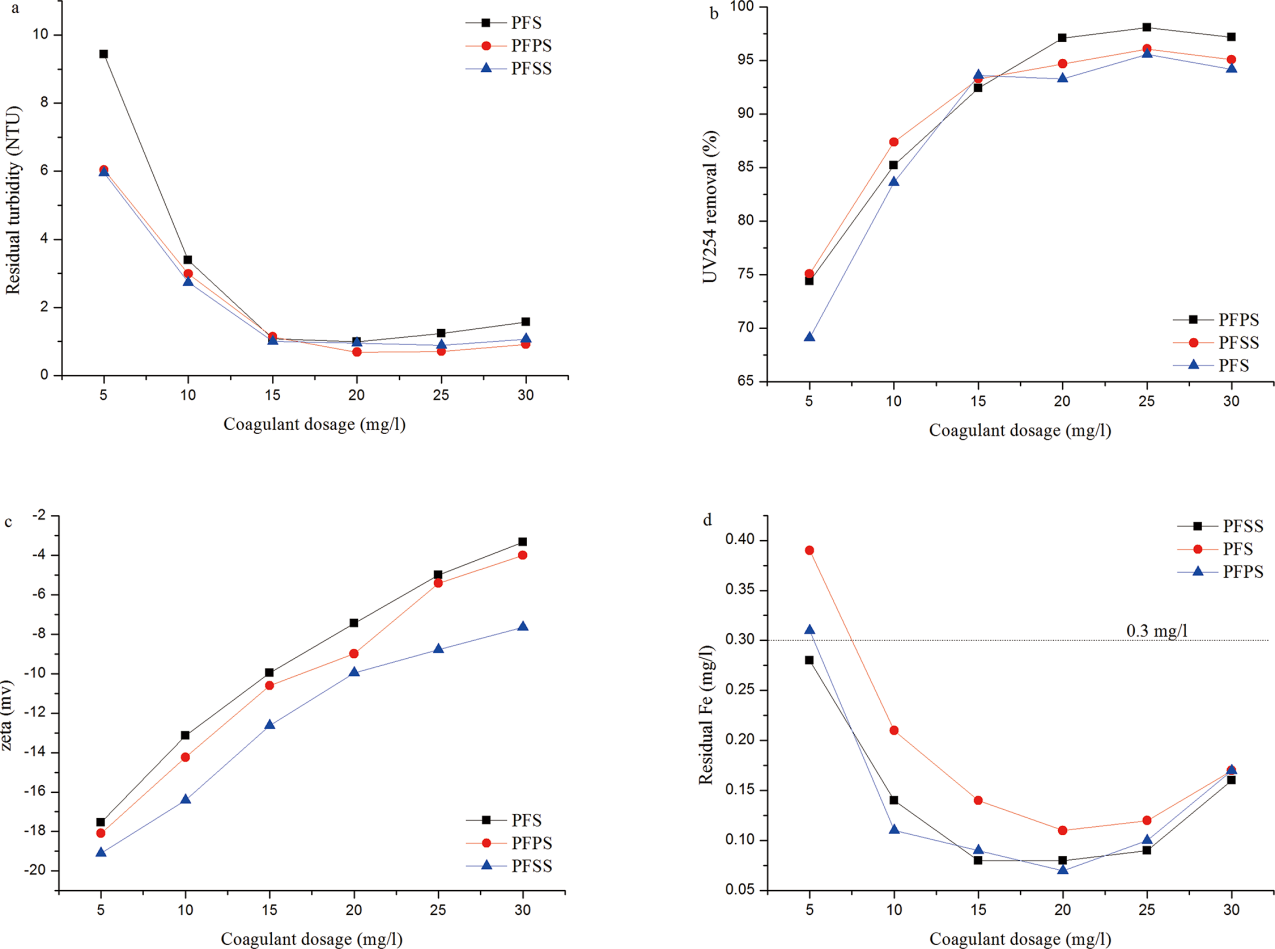
Effect of coagulant dosage on (a) residual turbidity, (b) UV_254_ removal efficiency, (c) zeta potential and (d) residual iron concentration.

Fig 6 shows the comparative performance of PFS, PFSS, and PFPS as they treated kaolin–humic acid suspensions at coagulant dosage between 5 and 30mg/l as Fe_2_O_3_. Fig 6(A) indicate where residual turbidity decreased with increasing coagulant dosage from 5 to 15mg/l, reached a flat lower value around 0.8 NTU, then deteriorated at higher dosages. Notably, at lower dosages, PFSS showed superior coagulation efficiency, while at higher dosages, PFPS behaviors were more favorable. PFS showed inferior performance throughout the full coagulant dosage scale. The efficiency of all three coagulants in removing organic matter in terms of UV_254_ indicates similar removal efficiency as turbidity reduction. Fig 6(C) and 6(D) shows the variance of zeta potential and residual soluble iron concentration as a function of specific coagulant and dosage. Similar to residual turbidity, the residual Fe decreased and then slightly increased as coagulant dosage increased. The minimum residual concentration (obtained from 15 to 25mg/l) was lower than 0.1 mg/l, and the value lower than 0.3mg/l, in most cases, met Chinese drinking water standards (GB 5749–2006). The colloid zeta potential in the beaker solution continued decreasing from -22.4 mv to -3.1 mv as dosage increased, reaching negative values, even, at maximum dosage. The zeta potential of PFS proved higher than others, and PFSS showed the lowest zeta potential over the full coagulant scale.

The introduction of PO_4_^3-^ and SiO_3_^2-^ anion modifiers weakened the charge neutralization ability of the reagent, one of the primary mechanisms of the coagulation process [24, 39]. The mechanisms which most closely represent the destabilization of colloids include electric-double layer compression, adsorption and charge neutralization, entrapment, and interparticle bridging [40]. Superior turbidity reduction and organic removal efficiency in the coagulation process suggest factors other than adsorption–charge neutralization; instead, adsorption–bridge formation is predominant. Considering alkali titration and species distribution, the introduction of PO_4_^3−^ and SiO_3_^2−^displaced the hydroxyl in the PFS sample and formed a complex, multi-core, medium-weight polymer, owing to the bridge effect between the iron atoms [18]. When the coagulant was added to the solution, the multi-core polymer hydrolyzed into a multi-chain polymer, attaching polymer chains to the surface of particles at several points by adsorption, eventually linking particles together to form an aggregate. Further adsorption-bridging of the aggregate resulted in larger-sized flocs (311.69, 319.56 and 271.21 μm for PFPS, PFSS and PFS, respectively.) and formation of sediment, thus raising the coagulation efficiency.

Compared to PFPS, the superior coagulation performance of PFSS at lower dosage (lower than 15 mg/l) may be ascribed to that the polysilicic acid could adsorb and precipitate a part of impurity, thus resulting in more turbidity reduction and UV_254_ removal. At higher dosage (higher than 15 mg/l), the inferior coagulation performance of PFSS can be attributed to the following: 1) strong electronegativity of SiO_3_^2-^ lowering the charge neutralization capability of coagulants, 2) gelatinization of polysilicic acid eliminating the coagulation effect, and 3) weak complex ability of Fe^3+^ and SiO_3_^2-^ preventing higher multi-core polymer [11, 41]. On the contrary, PFPS demonstrated superior performance in stability and coagulation efficiency.

### Modifier and pH vs. coagulation-flocculation performance

The influence of pH on the coagulation efficiency of three coagulants was examined in the pH range of 4.5–10.5 based on previous studies [42, 43].The coagulant dosage in this experiment was fixed at 20 mg Fe_2_O_3_/l.

**Fig 7:**
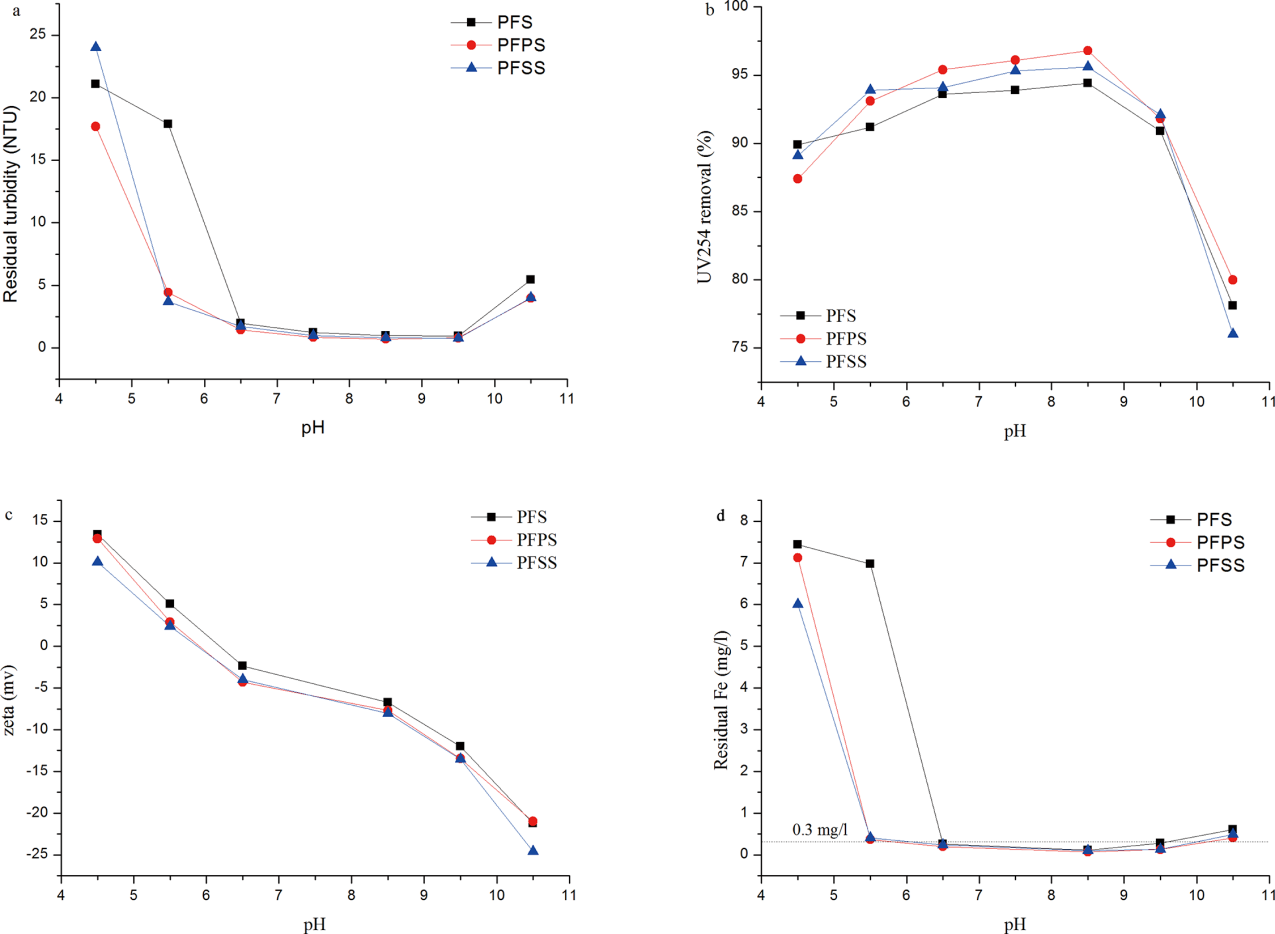
Effect of pH on (a) residual turbidity, (b) UV_254_ removal efficiency, (c) zeta potential and (d) residual iron concentration.

Fig 7(A) shows the residual turbidity curve decreased or increased with changing pH value. The optimal range was 6.5 to 9.5. Similar to turbidity reduction, the organic removal efficiency increased at first and then decreased within the pH range examined. Remarkably, residual turbidity shot up and even exceeded initial turbidity, and UV_254_ removal efficiency reached 87% at pH 4.5. Fig 7(C) depicts the zeta potential of colloidal particles reversed from positive to negative from 12.6 mv to -25.1 mv. Lower pH does not successfully allow for complete hydrolysis of Fe-based coagulants, so colloidal particles did not gather into sediment by adsorption/charge neutralization mechanism in a positive-charged solution system. Partially hydrolyzed coagulant adsorbed low-solubility humic acid (humic acid solubility was smaller in lower pH), resulting in decreased UV_254_ after filtration through the 0.45-μm membrane. Poor UV_254_ removal efficiency at higher pH can be assigned to the high solubility of humic acid in alkali environments, where it failed to be adsorbed to the surface of the coagulant’s hydrocolloids. Fig 7(D) shows where solubility iron concentration soared at lower pH, 4.5 and 5.5, and dropped sharply at with the increase of pH. The minimum concentration appeared from pH 7.5 to 9.5, and concentration began to increase above 9.5. At high pH value, a lot of negative charge species such that Fe(OH)_4_^-^ formed by uncontrolled hydration of coagulants, and kept suspended state with negative charged impurity colloids due to the electrostatic repulsion [1, 44].

PFS was more susceptible to pH value than the modified coagulants. The main reason for the enhanced performance of modified coagulants was the polymer effect [45]. The introduction of PO_4_^3−^ and SiO_3_^2−^ modifiers facilitated the hydrolytic polymerization of coagulants, resulting in high-molecular polymers [1, 18, 46]. When high-molecular polymer coagulants were added to the water samples, hydrocolloids were able to interact with suspended particles easily and aggregate into larger flocs. The residual turbidity of PFSS and PFPS at pH of 5.5 was around 4, while the residual turbidity of PFS was higher than the initial turbidity. This suggests that larger flocs in the modified coagulants sedimented perfectly, even in lower pH. The UV_254_ removal efficiency and residual turbidity of PFPS reached 96.3% and 0.69 at pH 8.5, respectively, far superior to PFSS.

Residual iron was at a similar level as residual turbidity. The lowest values of PFPS, PFSS, and PFS were 0.07, 0.1, and 0.1mg/l, respectively, in the pH of 8.5. The gap between PFSS and PFPS can be explained by that more polymer species in PFPS forming large and dense flocs, which more easily sedimented during the coagulation process [17].

### Modifier and temperature vs. coagulation-flocculation performance

**Fig 8:**
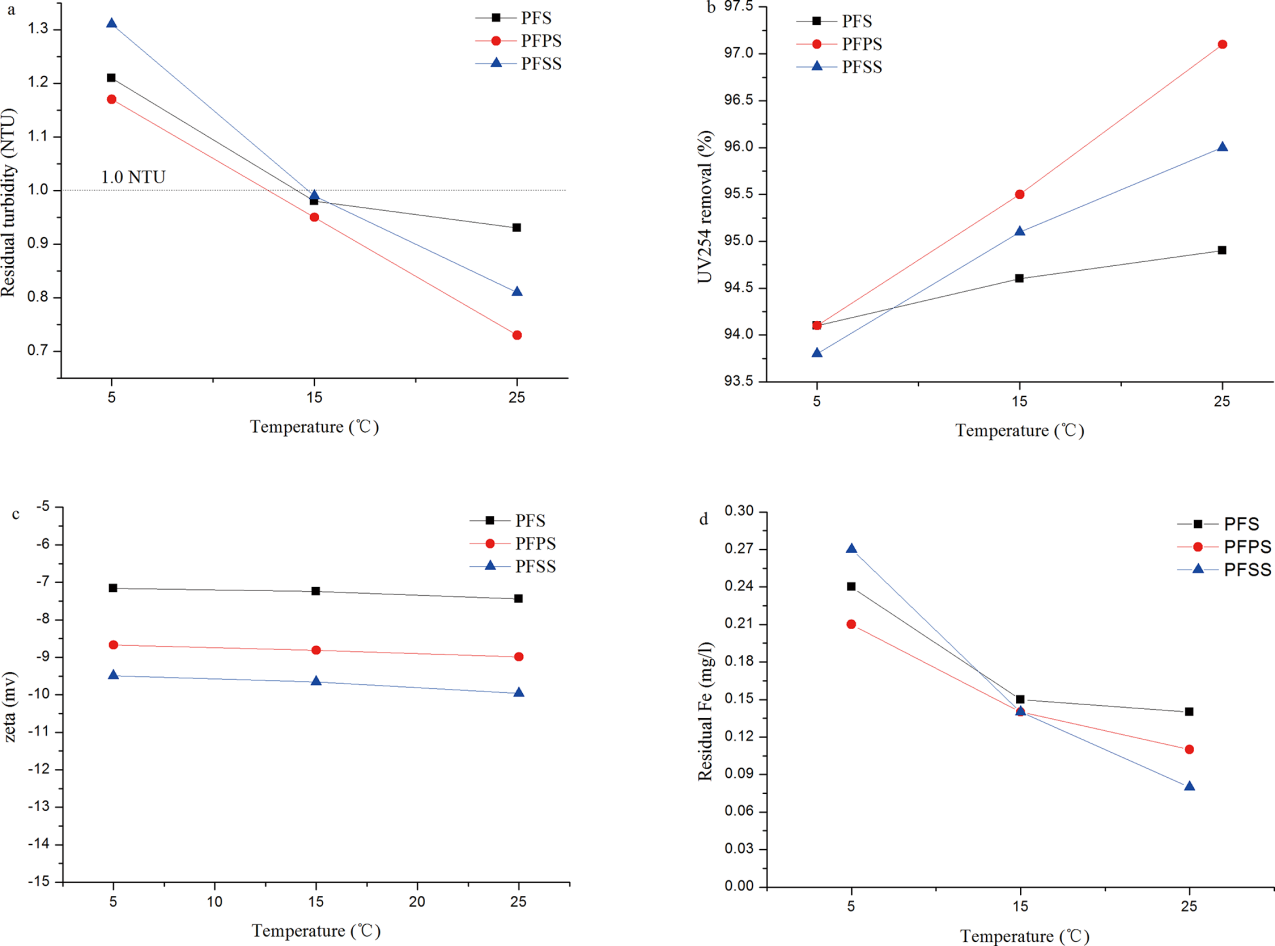
Effect of temperature on (a) residual turbidity, (b) UV_254_ removal efficiency, (c) zeta potential and (d) residual iron concentration.

Temperature, as established by many previous researchers, is closely related to fluid viscosity, particle-particle interactions, metal salt hydrolysis, adsorption, and precipitation rates [47–49]. To study the effects of temperature on the coagulant samples, jar tests were conducted at 5°C, 15°C, and 25°C. Results are illustrated in Fig 8.

The diagram below shows a small but visible gap in residual turbidity and UV_254_ reduction efficiency between the three water sample temperatures. Turbidity was reduced from 1.2 NTU to around 0.9 NTU, and UV_254_ removal efficiency increased from 93.8% to 96%. It is worth noting that the gap in coagulation efficiency was small at lower temperatures, but enlarged at high temperatures. Fig 8(D) shows the residual iron in 5°C was higher than in 25°C, and that the concentration of PFSS in 5°C was the highest. Elevated residual turbidity and iron concentration may have been due to the fact that polysilicic acid does not readily precipitate in lower temperatures, and that the Fe^3+^ complex in SiO_3_^2-^ had re-released into the solution. The turbidity reduction and UV_254_ removal efficiency of PFPS were best, out of the three coagulants, which demonstrate PFPS as more suitable for low-temperature water treatment.

### Modifier vs. floc properties and mechanism

**Fig 9:**
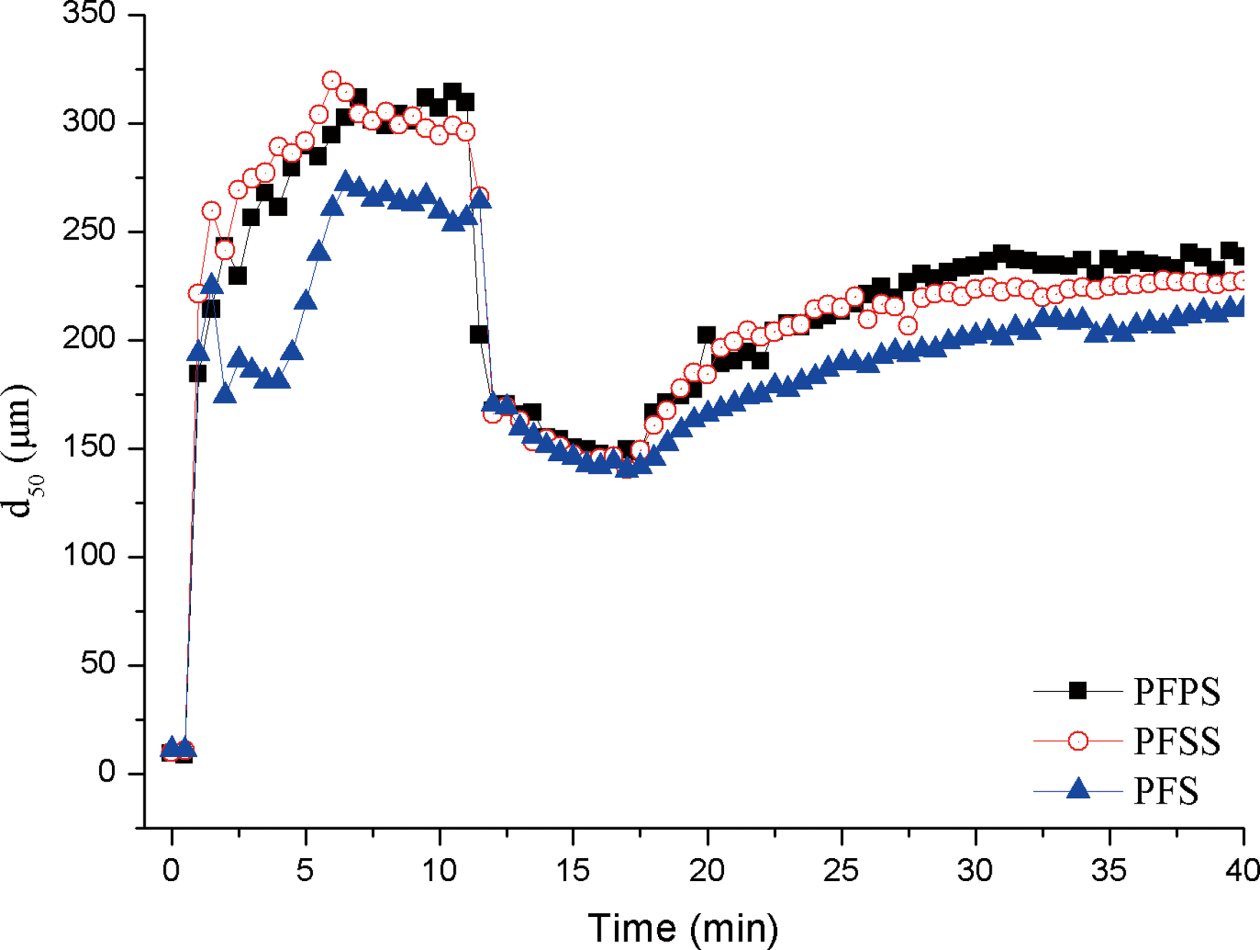
The growth, breakage and regrowth profile of three coagulant versus coagulation time and shear rate.

In order to investigate the floc growth rate and rates of floc breakage/regrowth under high shear, a series of comparative jar tests, which described changes in floc size as a function of coagulation time and shear rate, were conducted and exhibited in Fig 9. According to the coagulation experiment result in Fig 6, the optimum coagulation behaviors were achieved at the dosage range of 15 to 25 mg Fe_2_O_3_/l. Thus, the coagulation process was performed at a dosage of 20 mg Fe_2_O_3_/l for all three samples. The d_50_ value indicates that 50% of flocs were sized 0-d_50_ (The equivalent volumetric diameter of d_10_ and d_90_ is similar to d_50_.) The median diameter (d_50_) was adopted as the representative floc size. Similar trends were observed in d_10_ and d_90_. The growth, strength, and recovery factor of d_50_ are summarized in Table 1.

The profiles in Fig 9 show that variations in floc size were almost the same for all three Fe-based coagulants. In the steady state of floc growth, floc size spiked at the end of rapid stirring, followed by a slower growth rate until reaching the largest floc size of 314.5, 319.56, and 272.21μm in PFPS, PFSS, and PFS, respectively. Table 1 shows where rates in the growth stage fell into the following order: PFSS>PFPS>PFS. After about 6 min of slow stirring, PFSS and PFS floc size began to shrink slightly as stirring continued. Colloidal surface charge was also measured as plotted in Fig 6, where the zeta potential of three coagulants fell into PFS>PFPS>PFSS order. Larger floc size and lower surface charge suggest that electrostatic neutralization was not the dominant mechanism in coagulation process, instead, bigger flocs were mainly due to adsorption and entrapment [27, 40].

During preparation of PFPS, PO_4_^3-^ reacted with Fe^3+^ to produce chemical Fe-P bond which resulted in increased polymerization. Once coagulant was added to the solution and stirred, dispersion and hydrolysis occurred quickly, and positive polymeric colloids decreased the negative charges on the solution’s colloidal surfaces and adsorbed into microflocs. During the floc growth phase, the final floc aggregate and tails of the coagulant extended from the flocs and attached to other flocs to form a bridging effect, in which the sweeping and enmeshment of microflocs onto Fe(III) polymeric colloid aggregated the microflocs. The coagulation process was similar during PFS preparation as PFPS and PFSS; though the charge neutralization capability was higher in PFS than the other two, the lower polymer degree resulted in finer flocs, which are ultimately adverse to the coagulation process.

Floc breakage and regrowth under high shear was investigated, as well. The strength factors and recovery factors of the samples are displayed in Table 3, in order, PFS>PFPS>PFSS and PFS = PFPS>PFSS, respectively. Fig 9 shows where floc size decreased sharply when stir speed increased from 40 rpm to 250 rpm, reaching minimal size near 150 μm. The floc size between breakage phases was almost the same, with only minor discrepancies between the three sample coagulants.

**Table 3:** Growth, strength and recovery factors of flocs for different coagulants.

Coagulant	Growth rate	Strength factor	Recovery factor
PFPS	51.94	0.47	0.55
PFSS	63.91	0.45	0.47
PFS	49.49	0.53	0.55

Previous studies have reported that larger floc size leads to weaker charge neutrality, entrapment, and adsorption of colloids [50]. Conversely, shorter distance between the Fe-core colloids and solution colloid particles strengthens the electrostatic adsorption force of finer flocs. Overall floc strength thus corresponds to the size and strength of floc interior bonds. Flocs break if the stress applied to their surface is larger than the bonding strength within them, whereas at smaller particle sizes, increased shear does not break bonds through electrostatic patch mechanisms between Fe core polymers and negative particles.

Floc recoverability at different rates was also observed in the samples, all at relatively slow velocities. As shown in Fig 9, the flocs do not regrow to their initial sizes at stirring time over 17 min. The recovery factors of PFPS, PFSS, and PFS in kaolin humic acid water treatment were 0.55, 0.47, and 0.55, respectively. To some extent, floc regrowth ability demonstrates internal floc bonding structure. Previous research suggests that the best recovery factor is achieved when colloid surface charge is close to the isoelectric point [51]. Higher zeta potential in PFS resulted in higher recovery factor supported the point. Whereas, the lower zeta potential and higher recovery factor in PFPS indicated that the flocs were not dominated by single charge neutralization, but instead were the result of a combination of strong adsorption, bridging, and charge neutralization. Adsorption, sweep and enmeshment were also important factors affecting floc recovery. The main floc recovery mechanism was different in PFPS opposed to PFSS—in PFSS, recovery was facilitated by the adsorption between polysilicic acid and solution particles, while sweeping and entrapment/adsorption resulted from larger polymer colloids of the Fe-P chemical bonds induced by enhanced recoverability.

### Comparison with commercial coagulants

**Fig 10:**
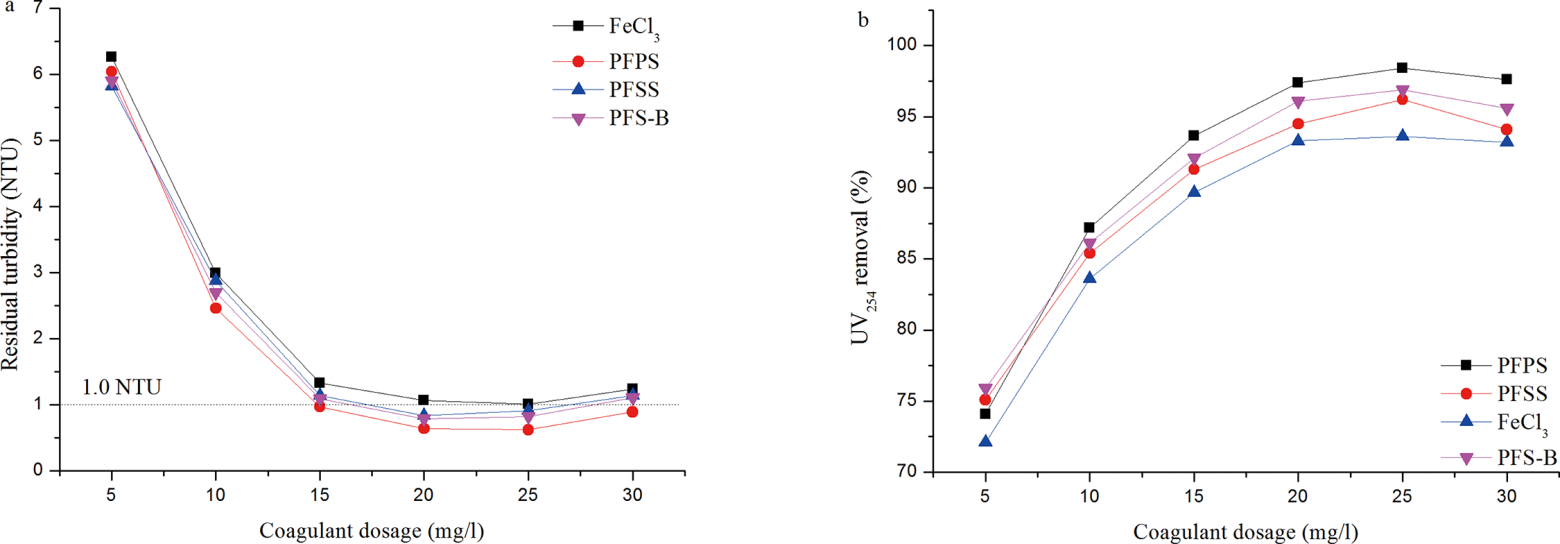
The comparison of PFS-B, FeCl_3_, PFSS and PFPS on (a) Turbidity reduction and (b) UV_254_ removal.

In order to study the effect of modifier on the improvement of coagulation efficiency, a series comparison experiments with commercial coagulants were conducted. Two commercial coagulants, Polyferric sulfate (denoted as PFS-B) and (FeCl_3_) Ferric chloride, which were obtained from Lanjie Tap Water Company (Chongqing, China) and Chengdu jin tai chemical co., LTD (Chengdu, China), respectively. Fig 10 shows the coagulation efficiency of four coagulants in turbidity reduction and UV_254_ removal with the dosage from 5 to 30 mg Fe_2_O_3_/l. The result illustrated that PFPS performed superior coagulation efficiency at the dosage above 10 mg/l, and the FeCl_3_ performed worst at full range of dosage. It is noteworthy that the turbidity reduction and UV_254_ removal of commercial PFS-B is superior to PFSS within the dosage investigation range, which demonstrated that the improvement by addition of SiO_3_^2-^ is limited. Whereas the experiment results indicate that compared with commercial PFS, the introduction of PO_4_^3-^ modifier enhanced the coagulation efficiency significantly.

## Conclusion

The most notable conclusions of this study can be summarized as follows.

- Three Fe-based coagulants, PFPS, PFSS, and PFS were prepared in the laboratory, as detailed above. X-ray diffraction showed that the introduction of PO_4_^3-^ produced complex, multi-core, large iron polymer, and that SiO_3_^2-^ addition synthetized the hexagonal crystal structure (SiO_2_)_x_ and NaFe^+3^(SiO_3_)_2_. Alkali titration curves indicated that more polymer species generated after introduction of PO_4_^3-^ and SiO_3_^2-^ modifiers. Ferron species analysis suggested, as per Fe_b_ content, that the stability of coagulants was in the following order: PFPS > PFSS >PFS.
- Jar tests demonstrated that introduction of PO_4_^3-^ and SiO_3_^2-^ anion modifiers enhanced overall coagulation performance. PFPS showed optimal coagulation behavior in terms of UV_254_ removal efficiency, residual turbidity, and residual iron concentration—observed at 96.3%, 0.69, and 0.07 mg/l, respectively.
- Experiments which tested coagulation behavior, and floc breakage and regrowth showed that PFPS displayed larger floc size, strength, and recovery. Results also showed that Fe-P chemical bonding enhanced the sweeping, entrapment, and adsorption ability of the PFPS polymer coagulant.
